# Enhancing the Gamma-Radiation-Shielding Properties of Gypsum–Lime–Waste Marble Mortars by Incorporating Micro- and Nano-PbO Particles

**DOI:** 10.3390/ma16041577

**Published:** 2023-02-13

**Authors:** Mahmoud T. Alabsy, Mona M. Gouda, Mahmoud I. Abbas, Shoaa Mofleh Al-Balawi, Ahmed M. El-Khatib

**Affiliations:** Physics Department, Faculty of Science, Alexandria University, Alexandria 21511, Egypt

**Keywords:** gypsum/lime mortar, micro PbO, nano PbO, linear attenuation coefficient, exposure buildup factor, radiation shielding

## Abstract

In the current study, the gamma-radiation-shielding characteristics of novel gypsum–lime–waste marble-based mortars reinforced with micro-PbO and nano-PbO powders were investigated. In total, seven mortar groups, including a control mortar (named GLM), were prepared. The other groups contained10, 20, and 30 wt.% of both micro-PbO and nano-PbO as a waste marble replacement. This study aimed to explore the effect of particle size and concentrations of PbO powders on the γ-ray-shielding capability of GLM mortars. For this purpose, an HPGe detector and five standard radioactive point sources (^241^Am, ^133^Ba, ^137^Cs, ^60^Co, and ^152^Eu) were employed to measure different shielding parameters, including the linear attenuation coefficient (μ), mass attenuation coefficient (μ_m_), mean free path (MFP), half-value layer (HVL), and tenth-value layer (TVL), for the prepared samples in the energy range between 59.53 keV to 1408.01 keV. On the basis of μ_m_ values, other significant shielding parameters such as effective atomic number (Z_eff_), effective electron density (N_eff_), equivalent atomic number (Z_eq_), and exposure buildup factor (EBF) were also computed to explore the potential usage of the proposed mortars as radiation protective materials. The results reported that the smallest HVL, TVL, and MPF, as well as the largest attenuation values, were obtained for mortars reinforced by nano-PbO compared to those containing micro-PbO. It can be concluded from the results that the mortar samples containing nano-PbO had a remarkably improved gamma-radiation-shielding ability. Thus, these mortars can be used for radiation shielding on walls in nuclear facilities to reduce the transmitted radiation dose.

## 1. Introduction

Over the past decades, ionizing radiation has received significant attention considering that radiation technology is used in numerous areas, such as in the medical and industrial fields, nuclear powerplants, and scientific research [1]. Although radiation from naturally occurring or artificial radioactive material is a powerful tool with great beneficial applicability in various areas, it carries biological and environmental hazardous risks. For example, gamma rays have enough penetrating power to pass through the human body, creating ionization and damaging tissue [2]. Therefore, protecting the nearby exposed people, equipment, and environment is essential to reduce the risk of radiation-related damage. For this reason, radiation protection has become a crucial subject in physics, attracting continued research [3]. The three main principles of radiation protection to control external radiation hazards are time, distance, and shielding [4]. The choice of material is essential in radiation protection; thus, recent studies have focused on selecting and designing effective shielding materials.

Lead and concrete are the well-known and most common traditional shielding materials used for protection from radiation hazards in radiation therapy facilities and nuclear reactors. Various dense materials such as tungsten, bismuth, copper, and steel are considered good shielding materials. However, different composites based on polymers, glasses, alloys, and mortars containing fillers, such as micro- and nanoparticles, have been extensively studied as alternative radiation-shielding materials which are suitable for the desired applications [5]. 

There is an increasing demand to develop new ecofriendly binders. Mortars differ from concrete in working consistencies, whereby they play a significant role in the masonry’s overall performance. Mortar is a composite material that acts anisotropically. The primary purpose of mortar in masonry is to bond masonry units to each other, while it is also used to uniformly distribute load over the lower brick or stone. Gypsum is a synthetic building material used in building ceilings and the interior coating of walls. Furthermore, in the medical field, it is used as a shield against low-energy X-rays in diagnostic radiology, such as dental X-rays and mammograms [6]. Theoretical calculations of the mass attenuation coefficients of cement, gypsum, and a mixture of gypsum and PbCO_3_ were reported using the MCNPX code [7]. Lime is a binder and stabilizer intended to ensure long-term strength gain. Sidhu et al. prepared fly ash–lime–gypsum (FaLG) bricks and studied their energy exposure buildup factors and dose parameters [8].

Stone played an essential role in the growth of the giant urban renaissance, as the use of marble in buildings increased significantly, which in turn led to an increase in the number of marble companies [9]. Marble is used for many purposes, such as walls, floors, decor, household items, and antiques [10]. Factories produce vast quantities of marble waste in the form of powder and pieces of irregular stones, leading to the pollution of the agricultural and animal environment. This marble waste can be utilized as a replacement for conventional natural coarse aggregate in concrete [11].

Lead oxide is an essential industrial compound with high density, low cost, and unique mechanical, electrical, and optical properties [12]. PbO is used to provide better shielding characteristics for high-energy gamma rays. Recently, El-Khatib et al. studied the radiation-shielding properties of polypropylene (pp) samples embedded with PbO micro- and nanoparticles [13]. The results showed that pp embedded with nano PbO provided better attenuation than that of pp embedded with micro-PbO at all studied energies. In another research, recycled high-density polyethylene (r-HDPE) loaded with both bulk PbO and PbO nanoparticles showed better shielding for γ-rays than r-HDPE itself [14]. Additionally, epoxy resins and unsaturated polyester resin were prepared as a hybrid composite, which was then reinforced by a PbO ceramic powder with a microparticle size or nanoparticle size at different concentrations [15]. The results demonstrated that, for the same thickness and the same concentration, nano-shields were more efficient than micro-shields. PbO has also been used in many studies as a filler to enhance the shielding properties when added to various materials such as glass [16] and building materials [17]. 

Gypsum and limestone are environmentally friendly materials, and it is worthwhile to study these materials as radiation shields. Gypsum has good insulation properties, is fire-resistant, and has a superior finish, in addition to being a remarkable building material that allows increasing surface durability and reducing the risk of environmental pollution. During the industrial processing of marble rocks such as cutting and polishing, about 20% to 30% of the material is wasted in the form of slurry and powder dust. Hence, the use of marble dust as a building material is economically valuable. The aim of this study was to develop an effective radiation-protective mortar based on gypsum, lime, and waste marble, reinforced with micro-PbO and nano-PbO powders. The effect of nanosized PbO on the γ-ray-shielding performance of gypsum mortar was also examined. The cross-section morphologies of the prepared samples were examined using a scanning electron microscope (SEM). The linear and mass attenuation coefficients, the half-value layer, the tenth-value layer, and the mean free path of the examined PbO/gypsum mortars were experimentally determined at different gamma-ray energies ranging from 59.53 keV to 1408.01 keV using an HPGe detector. Moreover, important shielding parameters, such as the effective atomic number (Zeff), electron density (Neff), equivalent atomic number (Zeq), and exposure buildup factors (EBFs) of the investigated samples, were theoretically computed to evaluate the shielding capability of the proposed mortars. 

## 2. Materials and Methods

### 2.1. Materials

Gypsum–lime–waste marble mortars were prepared from local commercial raw materials. The binder was made up of gypsum and lime and supplied by local Egyptian companies. In addition, waste marble in the form of powder collected from marble factories was used as aggregate. Table 1 displays the elemental analysis of gypsum, lime, and waste marble using energy-dispersive X-ray spectroscopy (EDX) analysis. Two different sizes of lead oxide particles were used. Bulk extra-pure yellow PbO microparticles (purity 99.0%) were obtained from Alpha Chemica Company, India, whereas PbO nanoparticles (purity 99.6%) were chemically prepared by the Nanotech Company in Egypt. Moreover, water was used in the mixing process to produce the mortar. 

### 2.2. Mixture Processing

The binder mixture (gypsum/lime) was fixed to 1:0.2 according to a previous study [18], and the mortar mixtures were prepared with a water-to-binder (w/b) fixed ratio of 0.5:1. In total, seven mortar groups, including a control mortar (named GLM), were designed as a reference mixture. The other groups contained 10, 20, and 30 wt.% of micro-PbO and nano-PbO as a waste marble replacement. The mortar mixtures groups were coded and prepared according to the mixing ratios listed in Table 2. For each of the seven mortar groups, all materials were weighed, according to the ratios shown in Table 2, using an electrical balance (Analytical Balance, GR200,Tokyo, Japan) with an accuracy of 0.0001 g, and then mixed in a standard rotating mixer for 10 min. Next, all the mortar samples were poured into cylindrical rubber molds with dimensions of 30 mm in diameter and 5 mm in height. Each set contained three specimens. The freshly mixed samples were placed in room conditions at 23 ± 1 °C for 7 days to harden. 

### 2.3. Gamma Attenuation Measurements

The experimental gamma-ray-shielding properties of the proposed samples were evaluated using a spectrometer consisting of a well-calibrated hyper-pure germanium cylindrical detector (HPGe) from Canberra (Model GC1520). The detector was connected to an electronic system (pre-amplifier, amplifier, high voltage source, and multichannel analyzer). The HPGe detector had a resolution of 1.85 keV at 1.33 MeV gamma-ray peak ^60^Co and relative efficiency of 15% in the energy range from 50 keV to 10 MeV [19]. To diminish the background radiations and minimize the X-ray interferences, the detector was placed in a lead block of 15 cm thick, with a copper lining on the inside. The energy of the incident gamma radiation was varied using five standard radioactive point sources: ^241^Am, ^133^Ba, ^152^Eu, ^137^Cs, and ^60^Co, purchased from Physikalisch-Technische Bundesanstalt PTB in Braunschweig and Berlin, Germany. Table 3 illustrates the values of the emitted energies associated with each standard point source.

**Figure 1:**
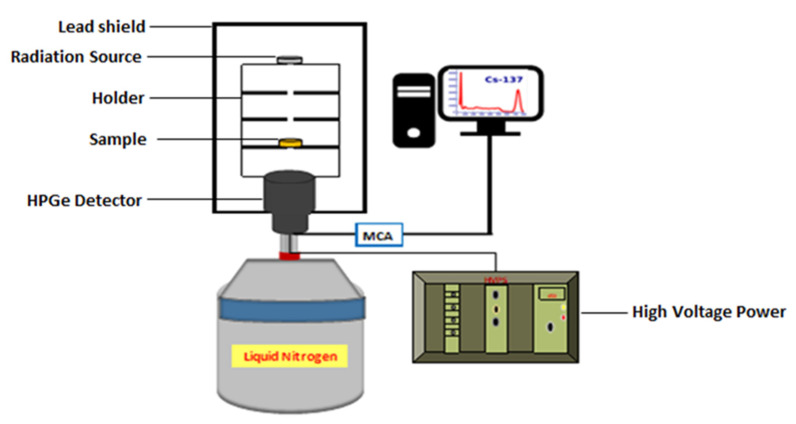
A schematic diagram of the experimental arrangement for gamma-ray measurements.

A schematic diagram of the experimental arrangement for gamma-ray measurements is depicted in Figure 1. Before starting the measurement process, a proper energy calibration procedure was carried out for the detector using three radioactive sources, ^241^Am (59.52 keV), ^137^Cs (661.66 keV), and ^60^Co (1173.23 and 1332.50 keV), to approximately cover the whole energy. The sample was placed between the source and the detector, as shown in Figure 1. The measurements were conducted for a sufficient time to obtain statistically significant Gaussian peaks so that the statistical uncertainty of the area under the photopeak was less than 1%. The electrical signal generated by the detector was amplified and then analyzed using Genie 2000 software by choosing a narrow region symmetric with respect to the centroid of the photo peak. The net area under the photo peak was computed, and then the count rate was calculated by dividing the obtained area by the acquisition time. The counting rate was calculated in the presence (I) and absence (I_0_) of the sample. The linear attenuation coefficient μ (cm^−1^) of each mortar sample was obtained at different γ-ray energies according to Beer–Lambert’s law given by Equation (1).

### 2.4. Theoretical Calculations 

Radiation shielding is based on the principle of attenuation, which is the ability to reduce radiation intensity through photoemission and scattering by a barrier material. When a gamma ray passes through an absorber material of thickness *x*, an exponential attenuation is observed in the intensity of the gamma radiation depending on the chemical content of this material according to Beer–Lambert’s law [20]:

(1) $$I=I_{0}e^{-\mu x},$$

where *I* is the transmitted intensity, *I_o_* is the incident intensity, *x* is the thickness of the absorbent (cm), and *μ* (cm^−1^) is the linear attenuation coefficient (LAC).

The half-value layer (HVL) and tenth-value layer (TVL) are defined as the thicknesses required to attenuate the incident photon intensity by factors of 1/2 and 1/10, respectively [21]:

(2) $$\mathrm{HVL}\;=\frac{\mathrm{Ln}2}{\mathrm{LAC}},$$

(3) $$\mathrm{TVL}=\frac{\mathrm{Ln}10}{\mathrm{LAC}}.$$

The mean free path (MFP) (cm) is the reciprocal of linear attenuation coefficient and denotes the average distance that a photon travels inside the sample without any interactions [1]. Moreover, MAC denotes the mass attenuation coefficient, which is measured by dividing LAC by the density (ρ) [22]:

(4) $$\mathrm{MAC}\;=\frac{LAC}{\rho }.$$

Other important shielding parameters can be derived from the mass attenuation coefficient, such as the effective atomic number (Z_eff_) and effective electron density (N_eff_). The Z_eff_ and N_eff_ parameters are fundamental in the field of nuclear radiation protection. Z_eff_ depends on the incident photon energy and is used to characterize the shielding properties of composites in terms of pure elements [23]. N_eff_ is the number of electrons per unit mass of the composite material measured in electrons/g. Z_eff_ and N_eff_ can be calculated from the following relationships [24]:

(5) $$Z_{eff}=\frac{\sum_{i}f_{i}A_{i}{\left(\frac{\mu }{\rho }\right)}_{i}}{\sum_{j\;}f_{i\;}\frac{A_{j}}{Z_{j}}{\left(\frac{\mu }{\rho }\right)}_{j}},$$

(6) $$N_{eff}=\frac{N_{A}Z_{eff}}{A},$$

where *f_i_*, *A_i_*, and *Z_i_* are the fractional abundance, the mass number, and the atomic number of the *i-*th constituent element in the composite material. $\langle A\rangle =\sum_{i}f_{i}A_{i}$ is the average atomic mass of the composite material, and *N_A_* is Avogadro’s number. 

The exposure buildup factor (EBF) is essential for radiation scattering studies and assessing shielding material performance. It is defined as the photon buildup factor in the air after penetration through a shielding material with high-energy photons. The EBFs for mortars were computed using G–P fitting parameters as mentioned in previously published studies, and this was achieved using the equivalent atomic number (Z_eq_), which is an energy-dependent parameter describing the properties of the investigated mortars in terms of their equivalent elements. Z_eq_ is defined by the following equation [25]:

(7) $$Z_{eq}=\frac{Z_{1}\left(\log R_{2}-\log R\right)+Z_{2}\left(\log R-\log R_{1}\right)}{\log R_{2}-\log R_{1}},$$

where *R*_1_ and *R*_2_ are the ratios (μ/ρ) _Compton_/(μ/ρ) _total_, which were obtained for mortar at a specified energy using the WinXCom program, corresponding to elements with atomic numbers *Z*_1_ and *Z*_2_, respectively. R is the ratio for the mortar at the specific energy, which lies between ratios *R*_1_ and *R*_2_.

The G–P fitting parameters such as (b, a, X_k_, d, and c) were then interpolated using the obtained *Z_eq_* values at a specific photon energy according to the interpolation formula [26]:

(8) $$C=\frac{C_{1}\left(\log Z_{2}-\log Z_{eq}\right)+C_{2}\left(\log Z_{eq}-\log Z_{1}\right)}{\log Z_{2}-\log Z_{1}},$$

where *C*_1_ and *C*_2_ indicate the G–P fitting parameters obtained from ANSI/ANS-6.4.3, the standard database, for *Z*_1_ and *Z*_2_, respectively.

The EBF values for the selected mortar samples were calculated in the energy range between 0.015 MeV to 15 MeV assisted by G–P fitting parameters using the following equations [27]:

(9) $$B\left(E,x\right)=1+\frac{b-1}{K-1}\;\left(K^{x}-1\right)\;,\;K\neq 1,$$

(10) $$B\left(E,x\right)=1+\left(b-1\right)x\;,\;K=1.$$

In these equations,

(11) $$K\left(E,x\right)=cx^{a}+d\frac{\tanh\left(x/X_{K}-2\right)-\tanh\left(-2\right)}{1-\tanh\left(-2\right)}\;\mathrm{for}\;x\leq \;40\;\mathrm{mfp},$$

where *E* is incident photon energy, and *x* is the mfp.

## 3. Results and Discussion

### 3.1. Microstructural Characterization

**Figure 2:**
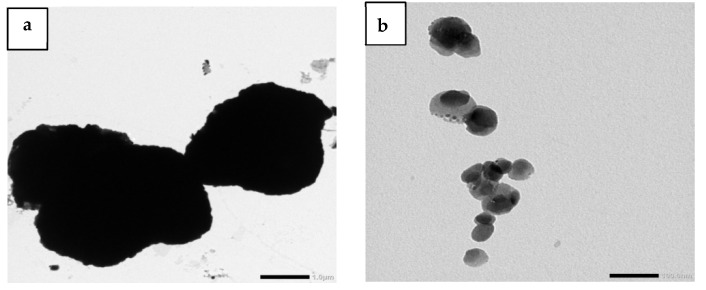
TEM images of (**a**) PbO microparticles, and (**b**) PbO nanoparticles.

By utilizing a high-resolution transmission electron microscope (TEM) (JEM 1400 Plus, JEOL, Tokyo, Japan) working at an accelerating voltage of 200 kV, the particle size of PbO micro- and nanoparticles was examined. The TEM micrographs of micro-PbO and nano-PbO particles are displayed in Figure 2. It is clear from Figure 2a that PbO microparticles had nonhomogeneous shapes with an average particle size on the order of 3 µm. On the other hand, Figure 2b demonstrates that PbO nanoparticles had a uniform shape with particle sizes ranging from 50 nm to 20 nm.

**Figure 3:**
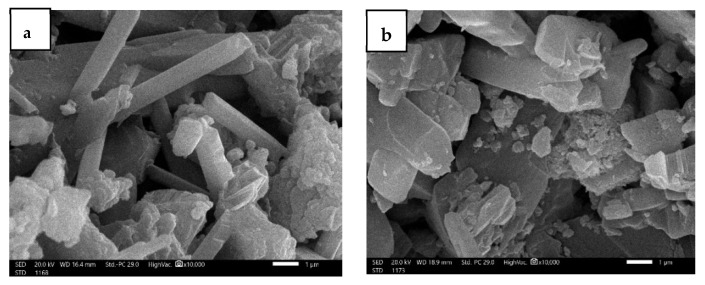

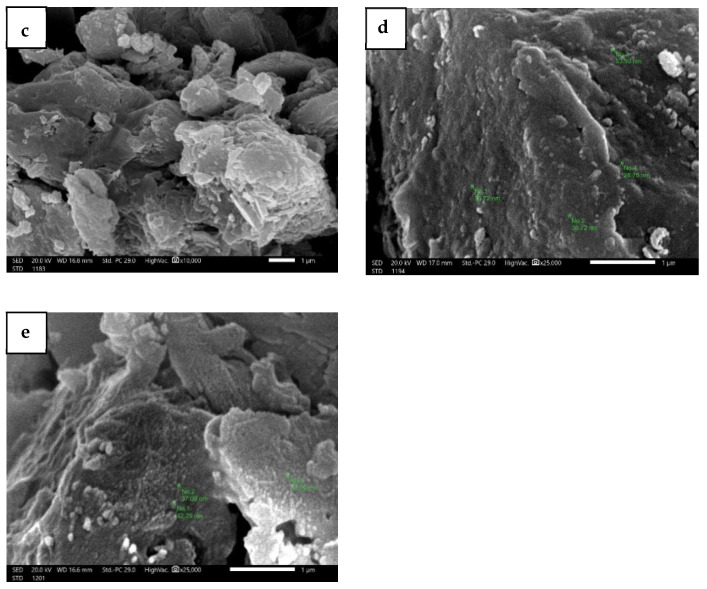
SEM images of the (**a**) GLM, (**b**) GLM-mPbO10, (**c**) GLM-mPbO30, (**d**) GLM-nPbO10, and (**e**) GLM-nPbO30 samples.

Furthermore, a scanning electron microscope (SEM) (JSM-6010LV, JEOL) was also employed to examine the cross-section morphologies of the prepared mortar samples. SEM samples had to be small and fixed with a double-coated carbon tap, which dissipated the electron beam charge and heat buildup. A fine layer of gold was used to cover the samples under a vacuum before the SEM treatment, using an ion sputtering coating device (JEOL-JFC-1100E). The SEM images were acquired at a magnification of 35,000× and a voltage of 20 kV. The SEM micrographs in Figure 3 clearly show the difference in morphology of the samples. The SEM image in Figure 3a depicts the morphology of pure mortar containing voids. Adding PbO microparticles at 10% and 30% concentrations showed an improvement in the mortar structure, as illustrated in in Figure 3b,c, respectively. The distribution of PbO nanoparticles with ratios of 10% (Figure 3d) and 30% (Figure 3e) was more uniform and homogenous than that of the microparticles. Because of their small size and characteristics, introducing PbO nanoparticles effectively increased the homogeneity inside the mortar, reduced the ratio of spaces in the sample, improved the radiation properties, and increased the attenuation coefficients.

### 3.2. Gamma-Ray-Shielding Characteristics

MAC is an essential basic parameter, which was used to study the radiation-shielding capability of the mortars under investigation. MAC was calculated experimentally using standard radioactive point sources that emit gamma-rays in the energy range between 59.53 keV and 1408.01 keV. The experimental results of MAC were compared to those calculated theoretically using the XCOM database [28], and they are listed in Table 4, along with their discrepancy (∆%). The discrepancy (∆%) between the experimental and XCOM values of MAC was calculated using Equation (12).

(12) $$\Delta \%=\frac{MAC_{exp}-MAC_{XCOM}}{MAC_{XCOM}}.$$

As can be noted from Table 4, the discrepancy varied in the range of −1.24% to 4.62% for GLM, −3.56% to 3.23% for GLM-mPbO10, −2.50% to 4.37% for GLM-mPbO20, and −6.25% to 2.13% for GLM-mPbO30 mortars. Thus, Table 4 confirms that the experimental results of MAC for all the investigated mortars reasonably matched those obtained theoretically from the XCOM database through all energy regions, confirming our experimental setup’s validity. As observed from the data analysis in Table 4, the MAC decreased sharply with increasing incident photon energy and increased significantly upon increasing the concentrations of PbO fillers in the GLM matrix. 

**Figure 4:**
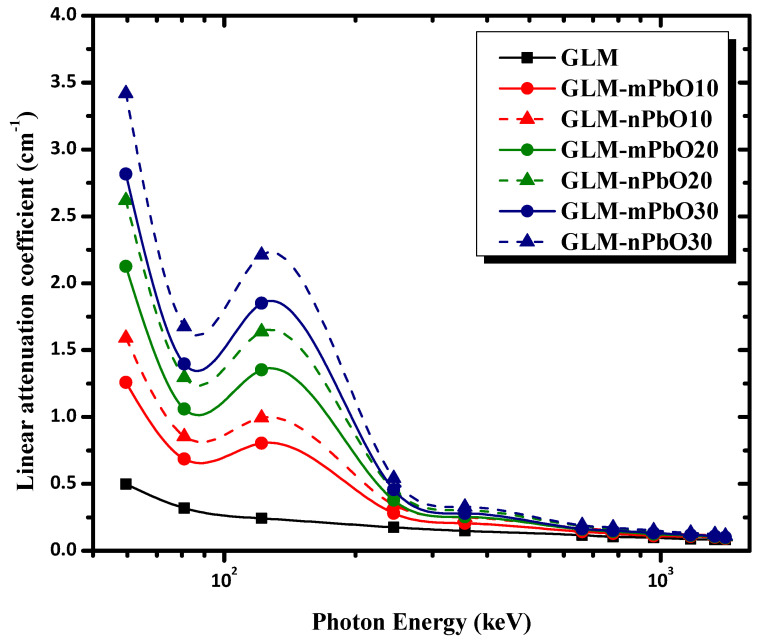
Linear attenuation coefficients for micro- and nano-PbO mortars as a function of photon energy.

The linear attenuation coefficients (LACs) were measured experimentally for the present mortar samples using a narrow beam of γ-ray transmission geometry, as presented in Figure 1, with the aid of Equation (1). The measured values of the LAC for the current micro- and nano-PbO/gypsum mortar samples versus incident photon energy in the range between 59.53 keV to 1408.01 keV are plotted in Figure 4. As can be noted from Figure 4, the presence of micro-PbO as a waste marble replacement in the gypsum mortar affected the shielding properties of the mixture. It is obvious from Figure 4 that the LAC values increased gradually with increasing concentrations of micro-PbO and decreased sharply with increasing incident photon energy. Furthermore, there was a sudden increase in the attenuation coefficient of photons at an energy of 121.78 keV. This sudden increase in attenuation was due to the appearance of the K absorption edge of Pb at 88 keV. 

The effect of replacing micro-PbO particles with nanosized PbO particles on the γ-ray-shielding performance of the gypsum mortars was also examined. The mortars reinforced by nano-PbO showed higher LAC values than those containing micro-PbO at the same particle concentration and photon energy, indicating the particle size’s impact on enhancing the shielding capability. That is, the mortars filled with nano-PbO had better gamma-radiation-shielding efficiency than those loaded with micro-PbO, which agrees with the findings published in the literature [13,14,24]. As the size of PbO particles decreased from micro- to nanoscale, the particles became uniformly dispersed over the entire area within the mortar, which increased the interaction probability between the incident photons and the nanoparticles, thus increasing the chance of the photons undergoing more scattering and absorption processes. In addition, the surface area of nanoparticles is higher than that of microparticles; therefore, the photons underwent several scattering processes until their energy became less than 200 keV, ultimately being absorbed through the photoelectric effect. Hence, PbO nanoparticles led to higher radiation absorption.

**Figure 5:**
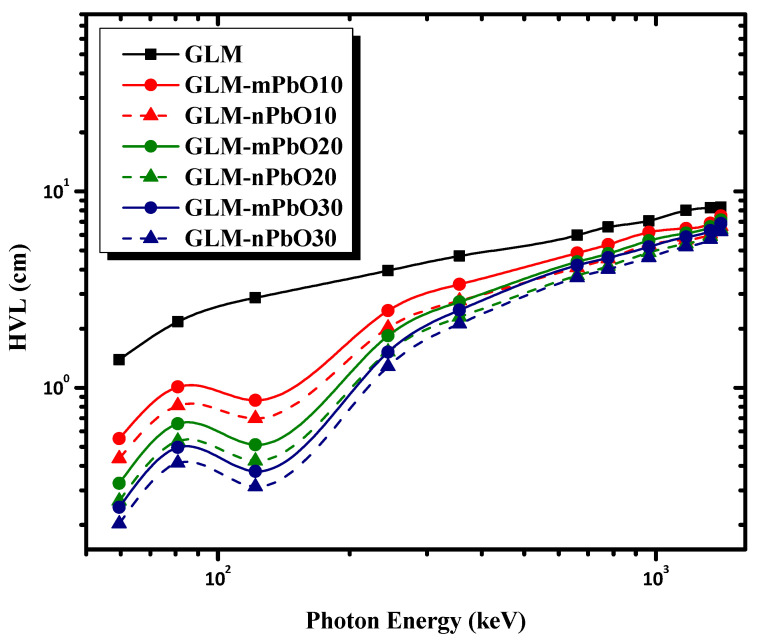
The variation of HVL as a function of photon energy for micro- and nano-PbO mortars.

**Figure 6:**
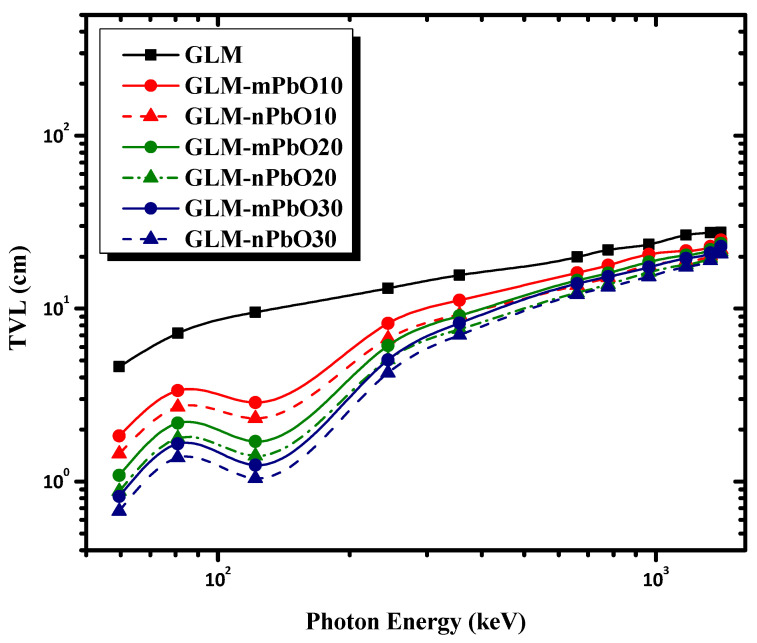
The variation of TVL as a function of photon energy for micro- and nano-PbO mortars.

**Figure 7:**
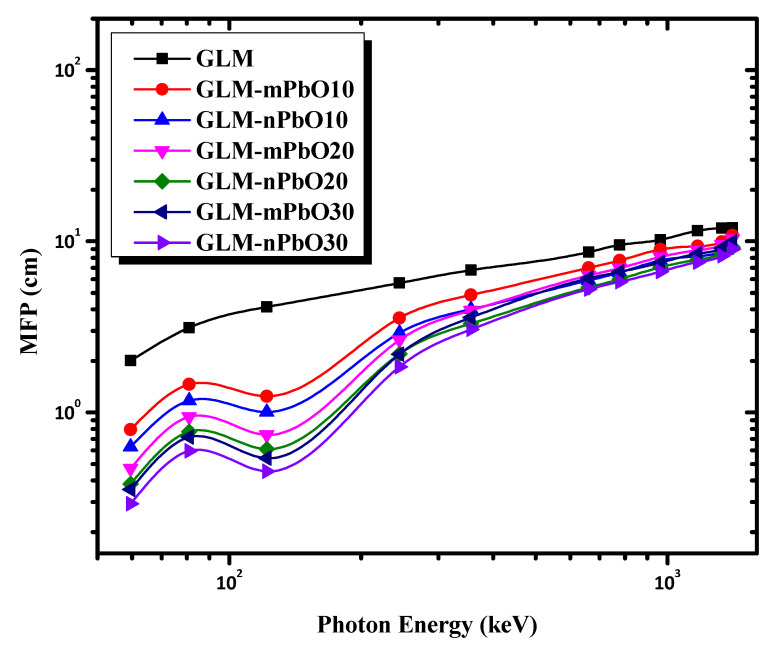
The variation of MFP as a function of photon energy for micro- and nano-PbO mortars.

The effectiveness of the shielding materials could be evaluated by measuring other important parameters such as HVL, TVL, and MFP. These parameters were used to check the shielding ability of the studied mortars. HVL and TVL were defined as the absorber thicknesses needed to reduce the incident γ-ray intensity to 50% and 10% of its initial value, respectively. The MFP represents the average distance that a photon traveled inside the sample without any interactions. Better shielding materials have low HVL, TVL, and MFP values. Figure 5, Figure 6 and Figure 7 show the variations of HVL, TVL, and MFP, respectively, as a function of photon energy for all the prepared mortars. As a general trend, HVL, TVL, and MPF increased with increasing the photon energy, emphasizing that higher-energy photons tended to penetrate through the mortar with greater ease than lower-energy photons. As an exception, there was a sudden drop in the HVL, TVL, and MPF values for all samples reinforced with PbO powder at 121.78 keV due to the K absorption edge of Pb at 88 keV. The results indicate that the control sample (GLM) had the greatest HVL, TVL, and MPF at all energies. Meanwhile, increasing the content of micro-PbO particles, characterized by a high atomic number and high density, decreased the HVL, TVL, and MPF values at the same photon energy. Figure 5, Figure 6 and Figure 7 demonstrate that the addition of PbO nanoparticles resulted in improved radiation protection, whereby HVL, TVL, and MPF values decreased as the particle size of PbO was reduced to nanoscale, leading to an improvement in the shielding effectiveness of the gypsum mortars. 

To explain the variance of shielding capability between mortars reinforced by nano-PbO and those containing micro-PbO, the relative increase rate *δ*% in LAC was calculated using Equation (13).

(13) $$\delta \%=\frac{MAC_{nano}-MAC_{micro}}{MAC_{micro}}.$$

**Figure 8:**
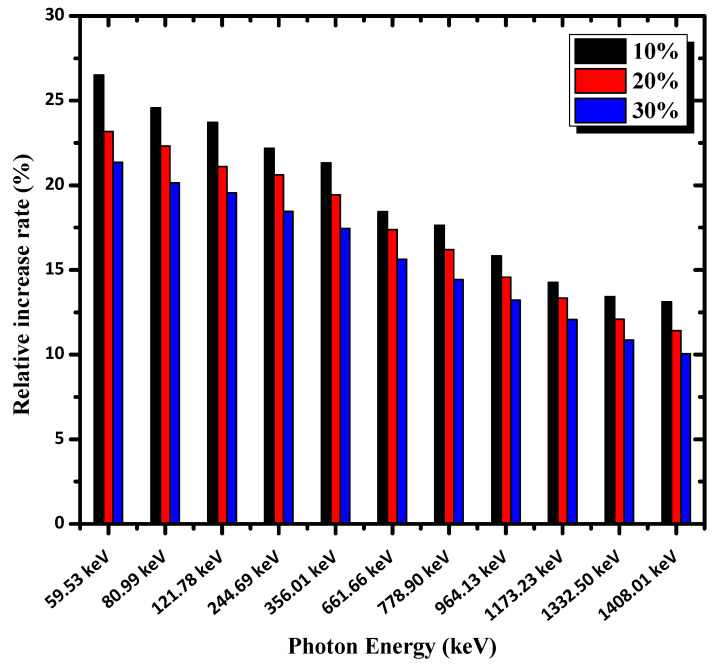
Relative increase rate between the micro- and nano-PbO samples as a function of photon energy.

Figure 8 displays the relative increase rate *δ* as a function of photon energy at different PbO particle concentrations. As can be seen from Figure 8, the value of *δ*% decreased as the photon energy increased. Moreover, at a specific photon energy, the value of *δ*% declined with the increase in PbO concentration. In other words, a higher concentration of PbO nanoparticles led to a lower relative increase rate. This behavior can be attributed due to the aggregation of nanoparticles at high filler concentrations [29]. Therefore, the functional role of nanoparticles was reduced, and the effect of particle size decreased at high filler loadings.

**Figure 9:**
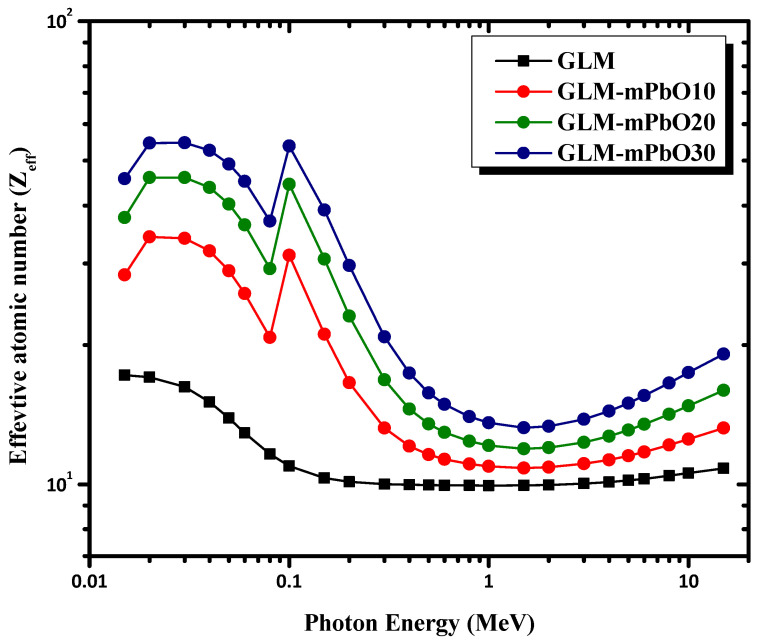
The Variation of the Z_eff_ with incident gamma-ray energy of mortars.

**Figure 10:**
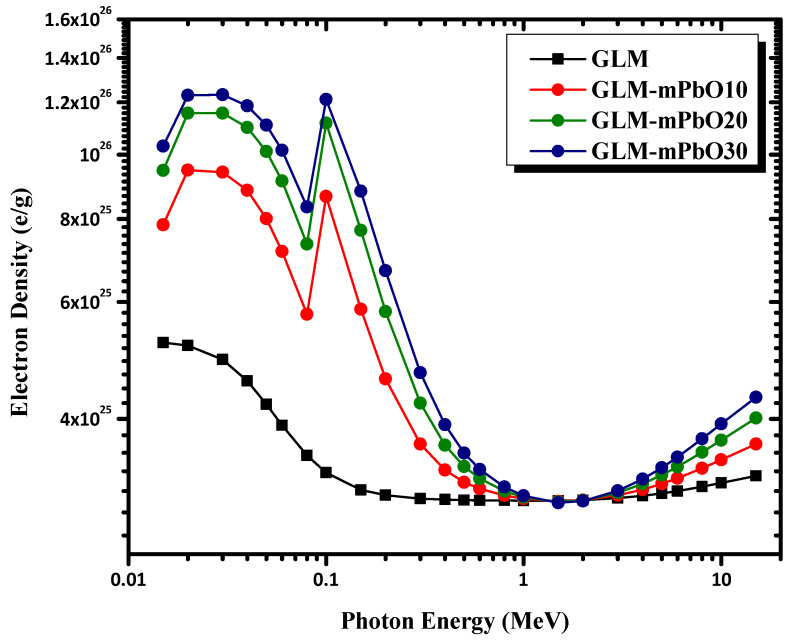
The Variation of the N_el_ with incident gamma-ray energy of mortars.

The variations of the effective atomic number (Z_eff_) and effective electron density (N_eff_) for total photon interactions of micro-PbO/gypsum mortars are shown in Figure 9 and Figure 10, respectively. Z_eff_ and N_eff_ for the selected mortars are plotted in the energy range 0.015–15 MeV. It can be observed that the Z_eff_ values depended on the incident photon energy and increased with increasing PbO concentration. Thus, values of Z_eff_ for GLM-mPbO30% samples were higher than those of the other samples in the low-energy range (0.015 MeV–0.2 MeV) due to the dominance of photoelectric absorption in this region. Therefore, GLM-mPbO30% samples could be considered better at shielding low-energy gamma-rays due to the high concentration of PbO, which could absorb the incident photons via the photoelectric mechanism. The highest Z_eff_ values found for mortars GLM-mPbO30%, GLM-mPbO20%, and GLM-mPbO10% were at E = 0.1 MeV due to the K absorption edge of Pb at 88 keV. However, at medium and high energy, Z_eff_ values rapidly decreased with increasing energy, where Compton scattering and pair production mechanisms became predominant. The dependence of N_eff_ on the incident photon energy is similar to that described for Z_eff_.

**Figure 11:**
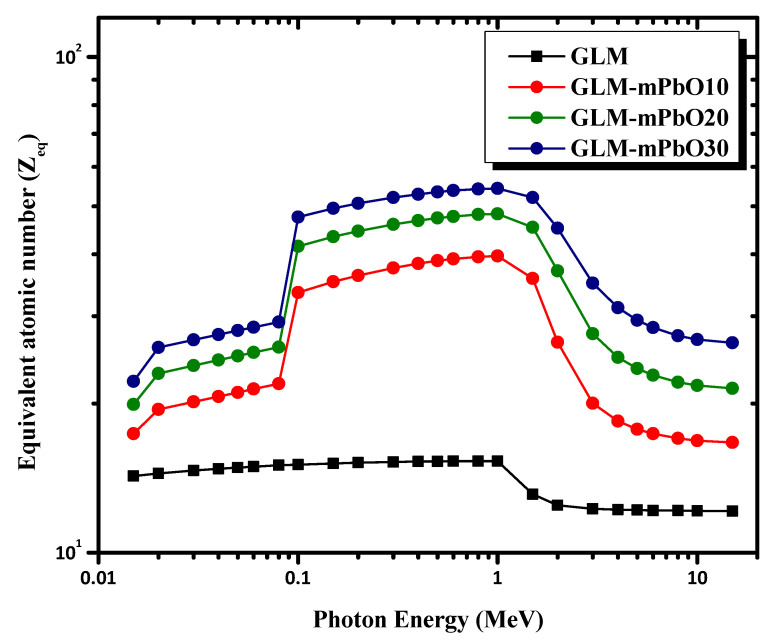
The variation of the Z_eq_ with incident gamma-ray energy of mortars.

The equivalent atomic number Z_eq_ was used to describe the shielding properties of the selected mortar in terms of equivalent elements, and it was also a parameter used in the EBF calculations. Z_eq_ was calculated in an energy range between 0.015 and 15 MeV, as depicted in Figure 11. In general, we can notice a decrease in the values of Z_eq_ in the low- and high-energy regions, whereas the values were high in the medium-energy region. This trend can be attributed to the dominance of Compton scattering in the medium-energy region. Moreover, the highest value of Z_eq_ was observed for the GLM-mPbO30% sample, whereas the lowest was observed for the GLM sample.

**Figure 12:**
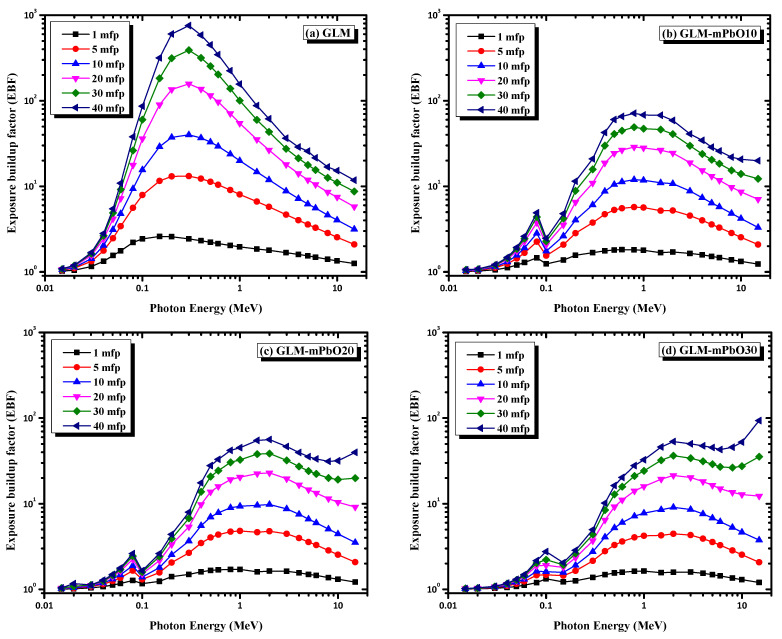
The energy buildup factor EBF dependence on energy at 1 mfp, 5 mfp, 10 mfp, 20 mfp, 30 mfp, and 40 mfp for mortars.

Figure 12 shows the EBF variance for all mortars with photon energy at different penetration depths. EBF was small at low photon energies, increased with increasing photon energy to reach a maximum value in the middle region, and decreased again with the rise in photon energy. This behavior was due to the dominance of the photoelectric effect and the pair production in the low and high regions, respectively, which resulted in a lower buildup factor, whereas the accumulation was higher in the middle zone due to the dominance of Compton scattering. The Compton process did not remove the photons. Nevertheless, it slowed them down due to the collision and scattering of photons with electrons, leading to a decrease in the kinetic energy of the photons and, thus, increasing the accumulation of photons in the middle region [30]. As reflected in Figure 11, the EBF values for all mortars were highest at 40 mfp, whereas the lowest values for EBF were recorded at 1 mfp. This behavior could be attributed to the generation of multiple photons due to increased interactions of photons at large penetration depths. Furthermore, values of the buildup factor for GLM were the highest among the selected samples. Upon increasing the content of the PbO in the gypsum matrix, the EBF values decreased, especially at low and moderate energies, while their maximums shifted to larger energies.

## 4. Conclusions

In this investigation, mortars were prepared from gypsum, lime, and waste marble and reinforced with micro-PbO and nano-PbO powders for their application in radiation shielding. According to the obtained results, the experimental values of the mass attenuation coefficients for micro-PbO/gypsum mortars matched well with those determined theoretically from the XCOM database. The morphological test was carried out using SEM for the prepared mortars. It was found that the addition of nanoparticles improved the morphological properties more than the addition of microparticles. The results showed that all the investigated shielding parameters depended on the incident photon energy and the chemical compositions of the mortars. The increase in content of micro-PbO increased the μ, Z_eff_, and N_eff_ values and reduced the HVL, TVL, and MFP values, leading to an improvement in the shielding effectiveness of the gypsum matrix. In addition, increasing the content of micro-PbO decreased the EBF values, especially at low and moderate energies. The results also demonstrated that the particle size of the PbO powder played a significant role in the shielding capability of the mortar. The mortars reinforced with nano-PbO had better gamma-radiation-shielding efficiency than those loaded with micro-PbO. We conclude that adding PbO nanoparticles improved the gamma-radiation-shielding ability of the gypsum mortars compared to the PbO microparticles.

## Figures and Tables

**Table 1:** Chemical composition as weight fraction (wt.%) of gypsum, lime, and waste marble.

Element	Chemical Composition (wt.%)		
	Gypsum	Lime	Waste Marble
C	3.80 ± 1.22	6.42 ± 0.35	11.54 ± 0.09
O	49.42 ± 0.21	51.56 ± 0.26	53.15 ± 0.53
Al	-	-	0.36 ± 0.15
Si	-	0.35 ± 0.19	0.70 ± 0.19
S	21.14 ± 0.73	-	-
Ca	25.65 ± 0.64	39.36 ± 0.21	34.04 ± 0.72
Na	-	0.34 ± 0.07	-
Mg	-	1 ± 0.03	0.21 ± 0.02
Cl	-	0.97 ± 0.12	-

**Table 2:** Mixing ratios of the mortar samples.

Sample Codes	Binder (1:0.2)	Aggregate			Water
		Waste Marble	Micro-PbO	Nano-PbO	
GLM	1	2	0	0	0.5
GLM-mPbO10	1	1.7	0.3	-	
GLM-mPbO20	1	1.4	0.6	-	
GLM-mPbO30	1	1.1	0.9	-	
GLM-nPbO10	1	1.7	-	0.3	
GLM-nPbO20	1	1.4	-	0.6	
GLM-nPbO30	1	1.1	-	0.9	

**Table 3:** Standard radioactive point sources parameters.

Nuclide	Energy (keV)
^241^Am	59.53
^137^Cs	661.66
^133^Ba	80.99
	356.01
^60^Co	1173.23
	1332.50
^152^Eu	121.78
	244.69
	778.90
	964.13
	1408.01

**Table 4:** Experimental and theoretical values of MAC for GLM, GLM-mPbO10, GLM-mPbO20, and GLM mPbO30 at different photon energies.

Sample	Energy (keV)	Density (g·cm^−3^)	MAC Micro PbO/GLM (cm^2·^g^−1^)		
			EXP.	XCOM	∆%
GLM	59.53	1.477	0.3378	0.3238	4.34%
	80.99		0.2166	0.2144	1.02%
	121.78		0.1634	0.1572	3.97%
	244.69		0.1188	0.1165	1.99%
	356.01		0.0999	0.1008	-0.86%
	661.66		0.0783	0.0774	1.21%
	778.9		0.0713	0.07219	−1.24%
	964.13		0.0663	0.06526	1.57%
	1173.23		0.0586	0.05923	−1.01%
	1332.5		0.0567	0.05553	2.17%
	1408.01		0.0565	0.05397	4.62%
GLM-mPbO10	59.53	1.761	0.7142	0.7224	−1.13%
	80.99		0.3892	0.3841	1.33%
	121.78		0.4567	0.4424	3.23%
	244.69		0.1593	0.1598	−0.28%
	356.01		0.1168	0.1162	0.53%
	661.66		0.0811	0.08019	1.20%
	778.9		0.0734	0.07356	−0.18%
	964.13		0.0634	0.06577	−3.56%
	1173.23		0.0607	0.0593	2.37%
	1332.5		0.0571	0.05547	2.89%
	1408.01		0.0525	0.05389	−2.63%
GLM-mPbO20	59.53	1.847	1.1514	1.121	2.71%
	80.99		0.5734	0.5538	3.54%
	121.78		0.7321	0.7276	0.62%
	244.69		0.2042	0.2032	0.48%
	356.01		0.1372	0.1315	4.37%
	661.66		0.0858	0.08264	3.78%
	778.9		0.0777	0.07494	3.67%
	964.13		0.0669	0.06628	0.96%
	1173.23		0.0612	0.05938	3.03%
	1332.5		0.0568	0.05541	2.50%
	1408.01		0.0525	0.05381	−2.50%
GLM-mPbO30	59.53	1.948	1.4457	1.5200	−4.89%
	80.99		0.7163	0.7234	−0.99%
	121.78		0.9497	1.0130	−6.25%
	244.69		0.2343	0.2465	−4.93%
	356.01		0.1432	0.1469	−2.50%
	661.66		0.0846	0.08509	−0.58%
	778.9		0.0773	0.07631	1.31%
	964.13		0.0682	0.0668	2.13%
	1173.23		0.0606	0.05945	1.98%
	1332.5		0.0563	0.05535	1.65%
	1408.01		0.0517	0.05373	−3.69%

## Data Availability

All data are available in the manuscript.
